# A Monte Carlo framework for missing wedge restoration and noise removal in cryo-electron tomography

**DOI:** 10.1016/j.yjsbx.2019.100013

**Published:** 2019-10-25

**Authors:** Emmanuel Moebel, Charles Kervrann

**Affiliations:** Inria - Centre de Rennes Bretagne Atlantique, Campus Universitaire de Beaulieu, 35042 Rennes, France; Institut Curie, PSL Research University, CNRS UMR 144, UPMC, 75005 Paris, France

**Keywords:** Cryo electron tomography, Missing wedge, Restoration, Inverse problems, Monte-Carlo simulation

## Abstract

We propose a statistical method to address an important issue in cryo-electron tomography image analysis: reduction of a high amount of noise and artifacts due to the presence of a missing wedge (MW) in the spectral domain. The method takes as an input a 3D tomogram derived from limited-angle tomography, and gives as an output a 3D denoised and artifact compensated volume. The artifact compensation is achieved by filling up the MW with meaningful information. To address this inverse problem, we compute a Minimum Mean Square Error (MMSE) estimator of the uncorrupted image. The underlying high-dimensional integral is computed by applying a dedicated Markov Chain Monte-Carlo (MCMC) sampling procedure based on the Metropolis-Hasting (MH) algorithm. The proposed MWR (Missing Wedge Restoration) algorithm can be used to enhance visualization or as a pre-processing step for image analysis, including segmentation and classification of macromolecules. Results are presented for both synthetic data and real 3D cryo-electron images.

## Highlights

• A computational approach to preserve the specimen integrity and reduce electron doses.
• Restoration of missing wedge in 3D cryo-electron tomography.
• Image denoising and artifact removal by using a Monte-Marlo simulation method.
• Comparisons to state-of-the-art algorithms and double-tilt acquisition.

## Graphical abstract

**Figure:**
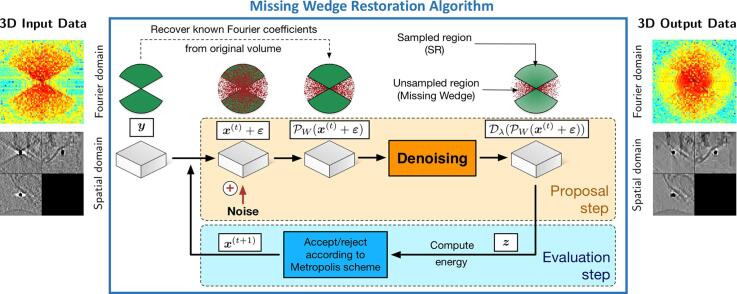

## 1 Introduction

Cryo-electron tomography (cryo-ET) is generally used to explore the structure of an entire cell and constitutes a rapidly growing field in biology. The particularity of cryo-ET is that it is able to produce three-dimensional views of vitrified samples at sub-nanometer resolution, which allows observing the structure of molecular complexes (e.g. ribosomes) in their physiological environment. Nevertheless, observation of highly resolved cellular mechanisms is challenging: i/ due to the low dose of electrons used to preserve specimen integrity during image acquisition, the amount of noise is very high; ii/ due to technical limitations of the microscope, complete tilting of the sample (90°) is impossible, resulting into a blind spot. As a consequence, projections are not available for a determined angle range, hence the term “limited angle tomography”.

**Fig. 1:**
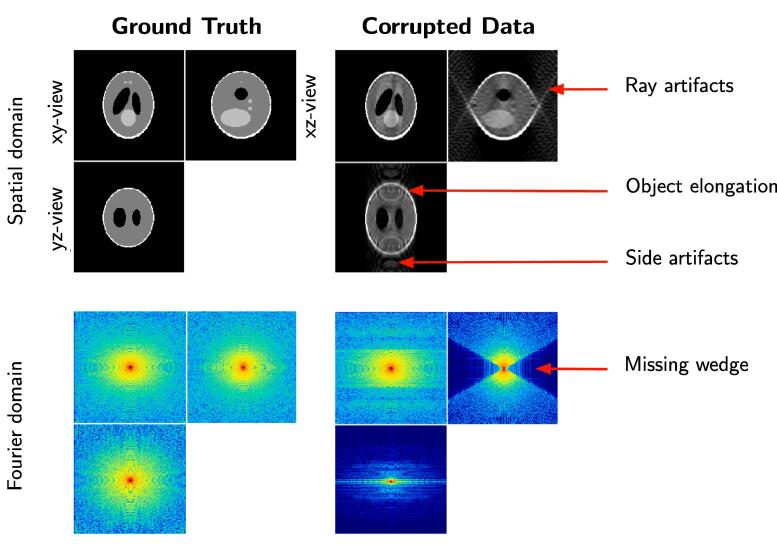
The effects of the missing wedge on the Shepp-Logan phantom. Left: uncorrupted 3D data displayed as ortho-slices (one central slice along each dimension). Right: data corrupted by the missing wedge. We display the data in spatial domain (top row) and Fourier domain (bottom row).

The blind spot is observable in Fourier domain, where the missing projections appear as a missing wedge (MW). This separates the Fourier spectrum into two regions: the sampled region (SR) and the unsampled regions (MW). The sharp transition between these two regions is responsible for a Gibbs-like phenomenon: ray- and side-artifacts emanate from high contrast objects (see Fig. 1), which can hide important structural features in the image. Another type of artifact arises from the incomplete angular sampling: objects appear elongated in the direction of the blind spot (see Fig. 1), in other words the data has an anisotropic resolution (e.g. linear features perpendicular to the tilt axis disappear). This elongation erases boundaries and makes it difficult to differentiate neighboring features. The quality of tomograms can be improved if sophisticated algorithms such as MBIR (Yan et al., 2019) are applied instead of conventional methods (e.g. WPB (Radermacher, 1992), SIRT (Gilbert, 1972)).

Filling up the MW with relevant data enables to potentially reduce or completely suppress these artifacts. Experimentally this can partially be achieved during data acquisition by using dual-axis tomography (Guesdon et al., 2013), where the sample is tilted with respect to the second axis. Consequently the blind spot is smaller and the MW becomes a missing pyramid, which results into a smaller missing spectrum. However dual-axis tomography is technically challenging and requires intensive post-processing in order to correct tilt and movement bias in the microscope. Another reconstruction approach consists in exploiting the symmetry of the underlying structure (Förster and Hegerl, 2007), but this can only be applicable to a limited number of biological objects (e.g. virus with either helical or icosahedral structure). Another common computational approach amounts to combining several hundred or thousands views of the same object, but with distinct blind spots. This so-called sub-tomogram averaging technique (Förster and Hegerl, 2007) is routinely used in cryo-ET, and is continuously improved for structure determination (Xu et al., 2018). To improve sub-tomogram averaging and compensate the remaining MW artifacts, tomographic reconstruction algorithms with dedicated regularization have also been proposed in (Paavolainen et al., 2014, Leary et al., 2013).

The objective of our work is to design a statistical approach for the problem of recovering missing Fourier coefficients from a single volume in the situation where low and high frequency coefficients are missing in a specific and large region of the 3D spectrum. A simple way of handling MW artifacts is described in (Kováčik et al., 2014), where a dedicated spectral filter is used to smooth out the transition between SR and MW; ray- and side-artifacts are reduced with this filter, but the object elongation remains in the resulting image. Inspired from Maggioni et al. (2013), we have rather investigated MCMC methods to compute a MMSE estimator based on any non-local image denoiser to recover the missing information. We show that our Monte-Carlo sampling algorithm performs as well as the iterative method (Maggioni et al., 2013) but converges faster. Nevertheless, our concept is more general since any denoising method can be applied, included denoising algorithms dedicated to cryo-ET images (Frangakis and Hegerl, 2001, Fernandez and Li, 2003, Darbon et al., 2008, Wei and Yin, 2010, Kervrann et al., 2008, Moreno et al., 2018). In this paper, we focus on the cryo-ET restoration problem but the proposed algorithm could be potentially used to address a large range of applications including medical and seismic imaging, and other inverse scattering problems.

  **Related work** We first focus on computational methods designed for spectrum restoration and Fourier coefficients recovering. Most of methods have been designed for 2D images and very few of them for 3D imaging. In general, the corruption process is supposed to be known and the artifacts observed in the input image, are due to a set of missing Fourier coefficients, well localized in the spectrum. First, several methods have been investigated to retrieve partially-missing phases of complex coefficients from modulus of coefficients in electron microscopy (Fienup, 1982) and time-frequency signal analysis (Krémé et al., 2018). Here, we focus on another special case which consists in extrapolating the band-limited spectrum of an image up to higher frequencies. Nevertheless, these problems are generally formulated as denoising problems with specific reconstruction constraints. For instance, Moisan (2001) and Guichard and Malgouyres (1998) investigated the Total Variation (TV) minimization to extend the band-limited spectrum of an image. In (Lauze et al., 2017), the authors combine TV minimization and positivity constraints to reduce noise and artifacts, providing an inpainting-like mechanism for the sinogram missing data in limited-angle tomography (see also (Frikel and Quinto, 2013, Sentosum et al., 2017)). The common objective is to create new frequencies while preserving discontinuities and details in the restored image. Instead of explicitly imposing some regularity (e.g. Total variation, or robust regularization (Geman and Reynolds, 1992, Charbonnier et al., 1997)) on the solution, another successful restoration approach consists in exploiting the spatial redundancy of the input image. In (Chambolle and Jalalzai, 2014), a non-local method was suggested in the framework of variational methods for image reconstruction. In this approach, a patchwise similarity measure based on atoms corresponding to pseudo Gabor filters is designed to compare corrupted regions. Meanwhile, Maggioni et al. (2013) adapted the concept of BM3D for recovering the missing spectrum applied to MRI imaging with very promising results on synthetic data. BM3D (Dabov et al., 2007) is a popular denoising algorithm which combines clustering of noisy patches, DCT-based transform and shrinkage operation to achieve the state-of-the-art results for several years. In our approach, we also focus on patch-based methods (Kervrann and Boulanger, 2008, Kindermann et al., 2005, Lou et al., 2010, Katkovnik et al., 2010, Pizarro et al., 2010, Milanfar, 2013, Sutour et al., 2014) to restore the input image corrupted by noise and non-linear transform. Indeed, it has been experimentally confirmed that the most competitive denoising methods are non-local and exploit self-similarities occurring at large distances in images, such as BM3D (Dabov et al., 2007), NL-Bayes (Lebrun et al., 2013), PLOW (Chatterjee and Milanfar, 2012), S-PLE (Wang and Morel, 2013), PEWA (Kervrann, 2014) and many other adaptative filters (Kervrann and Boulanger, 2006, Kervrann and Boulanger, 2007, Van De Ville and Kocher, 2009, Deledalle et al., 2009, Louchet and Moisan, 2011, Duval et al., 2011, Deledalle et al., 2012, Kervrann et al., 2014, Jin et al., 2017), inspired from the seminal N(on) L(ocal)-means algorithm (Buades et al., 2005).

To complete the brief overview of non-local methods, we mention that a noisy image can also be restored from a set of noisy or “clean” patches or a learned dictionary. The statistics of a training set of image patches serve then as priors for denoising (Elad and Aharon, 2006, Mairal et al., 2009, Zoran and Weiss, 2011). Another approach based multi-layer perceptron (MLP) exploiting a training set of noisy and noise-free patches was also able to achieve the state-of-the-art performance (Burger et al., 2012). Very recently, Buchholz et al. (2018) proposed to train content-aware restoration networks for denoising cryo-transmission electron microscopy data. While all these machine learning methods are attractive and powerful, computation is not always feasible in 3D because very large collection of 3D “clean” patches are required. In our study, we focus on unsupervised denoising methods since they are more flexible for real applications. They are less computationally demanding and are still competitive when compared to recent machine learning methods.

Our approach is mainly inspired from Maggioni et al. (2013), but can use any competitive denoising methods for restoring the Fourier coefficients. The method proposed by Maggioni et al. (2013) works by alternatively adding noise into the missing region and applying the BM4D algorithm which is the extension of BM3D (Dabov et al., 2007) to volumes. The authors interpret this iterative restoration method in the framework of compressed sensing with two information theory concepts in mind: *sparsity* of the signal in the transformed domain, and *incoherence* between the transform and the sampling matrix. Actually, BM4D does rely on a transform where the signal is sparse. Moreover, it is not clearly established that this transform is incoherent with the sampling matrix, defined by the support of the sampling region. Therefore, the proof of convergence is not clearly established, even though the authors presented convincing experimental results on synthetic images corrupted with white Gaussian noise. It remains unclear how the concept performs on experimental data and non Gaussian noise. To generalize this idea, we propose a statistical approach well-grounded in the Bayesian and MCMC framework and applied to challenging real data in cryo-ET. Our contributions are the following ones:

1. We present a MMSE estimator dedicated to the problem of MW restoration.
2. We propose an original Monte Carlo Markov Chain (MCMC) sampling procedure to efficiently compute the MMSE estimator.

**Paper organization** The remainder of the paper is organized as follows. In the next section, we present an overview of our computational method. In Section 3, we give the theoretical background and formulate the reconstruction problem as an inverse problem. We shortly describe the usual Bayesian approach to derive a MMSE estimator. Furthermore, a Monte-Carlo Markov Chains method based on the popular Metropolis-Hastings algorithm is proposed to compute the underlying high-dimensional integral. In Section 4, we adapt this general Bayesian framework for MW restoration. An original Metropolis-Hastings procedure is presented to explore the large space of admissible solutions and to select relevant samples. Section 5 presents the experimental results obtained on simulated and real data. We illustrate the potential of our MWR (Missing Wedge Restoration) algorithm with experiments on real cryo-tomogram images and we compare our iterative approach to several competitive algorithms.

## 2 Overview of the MW restoration algorithm

In this section, we present our concept for MW restoration. First, we formulate the problem and introduce notation. Second, we present the two-step algorithm which is embedded in a Monte-Carlo simulation framework.

### 2.1 Problem formulation and notation

Let us define a *n*-dimensional image $x:S\subset Z^{3}\to R$ assumed to be periodic and defined over a cubic domain $\Omega ={\left[0,1\right]}^{3}$ and n=|Ω|. The discrete Fourier transform of x={x(s),s∈S} is then as follows:

(1) $$Fx:k\to \underset{s\in S}{\sum }\exp\left(-2i\pi k\cdot s\right)x(s),$$

where *s* is the coordinate of point in spatial domain *S*. In our problem, one considers a corrupted image denoted y={y(s),s∈S} defined as

(2) $$y(s)=\underset{k\in \overset{‾}{W}}{\sum }\exp\left(2i\pi k\cdot s\right)Fx[k]$$

where $\overset{‾}{W}$ is the sampled spectral region (SR) where the Fourier coefficients Fx[k] are positive and non-zero. The region $\overset{‾}{W}$ is equivalent to the support of a binary mask $m\in {\left\{0,1\right\}}^{S}$ such as m[k]=1 if $k\in \overset{‾}{W}$ and 0 otherwise: $\overset{‾}{W}=\mathrm{supp}(m)$. The so-called missing wedge *W* is assumed to be symmetric with respect to the origin as illustrated in Fig. 1(bottom right), and $S=W\cup \overset{‾}{W}$. In what follows, we assume that the clean image Fx is known over the region $\overset{‾}{W}$. Our objective is then to estimate x:S→R in the whole domain *S* such that

(3) $$\forall k\in \overset{‾}{W},\;\;Fx[k]=Fy[k]$$

and ∀s∈S,x(s)>0. In other words, the set of known Fourier coefficients will be preserved by the restoration procedure. The challenge is to recover the unknown set of low, middle and high frequencies in a large region in the spectrum. This amounts to applying an interpolation operator $\varphi_{W}$ to the spectrum of y to get an estimator x^ of x:

(4) x^=F-1∘φW∘Fy.

### 2.2 Principles of the iterative two step-algorithm

**Fig. 2:**
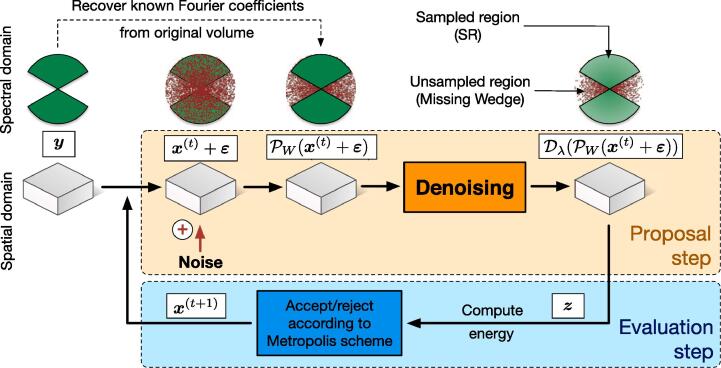
The MCMC method flowchart. The 1st icon row represents the data in the spectral domain, the 2nd in spatial domain.

Our computational method takes as input an noisy image y and iterates two steps as illustrated in Fig. 2. At the initialization (t=0), we set $x^{\left(0\right)}=y$:

• In the *proposal* step and at iteration *t*, white Gaussian noise ε is added to the current image $x^{\left(t-1\right)}$. The Fourier coefficients are then non-zero in the MW region *W*. We substitute the original Fourier coefficients in the SR region $\overset{‾}{W}$ to the noisy Fourier coefficients to preserve information. Formally, this amounts to applying an projection operator $P_{W}(\cdot )$ to the artificially noisy image. The objective is to randomly initialize the Fourier coefficients in the MW. A denoising algorithm is then used to efficiently remove the majority of the noise while preserving image structure. Due to its random nature, a small amount of the added noise will be close to the signal and is thus “saved” after denoising. The denoising algorithm D(·), which promotes smoothness while preserving contours, acts as a spatial regularizer. In summary, a candidate image $z=D(P_{W}(x^{\left(t-1\right)}+\varepsilon ))$ is obtained by applying an projection operator P in the Fourier domain and a denoising operator D in the spatial domain.
• In the second *evaluation* step, the candidate image z is compared and is potentially substituted to the current image $x^{\left(t-1\right)}$ depending on its ability to satisfies several constraints, including fidelity to the original input image z. A cost function (or energy) is used to accept/reject this candidate image with a certain probability established in the Metropolis-Hastings simulation framework as presented in the next section.

By repeating these two steps iteratively, we collect several hundreds of images $\left\{x^{\left(t\right)}\right\}$ to compute an average corresponding to the final restored image. The corresponding MWR (Missing Wedge Restoration) algorithm is actually able to diffuse information from SR into MW. It cannot retrieve unobserved data, but it merely makes the best statistical guess of what the missing data could be, based on what has been observed. The underlying algorithm is controlled by several parameters, especially the variance $\sigma_{\varepsilon }^{2}$ of noise added to the current image and constant at each iteration. More importantly, this iterative procedure will be successful provided the denoising algorithm is able to remove the artificial additive Gaussian noise. Any state-of-the art algorithms can be then applied, especially the BM3D (Dabov et al., 2007) and BM4D (Maggioni et al., 2013) algorithms. This stochastic procedure can be interpreted as a data-driven random search in a large space of possible images. In the next section, we present the theoretical background required to justify the rationale behind this concept, illustrated in Fig. 2.

## 3 Theoretical background

In this section, we describe the theoretical background in the Bayesian framework to justify our original computational method.

### 3.1 Bayesian estimator and Monte Carlo Markov Chain sampling

Solving inverse problems in image processing consists in estimating an unknown image x∈X given an image y∈Y. Different sources of distortion may cause damages on the ideal image, including noise, blur, and projections. In the Bayesian framework, the whole information once the data have been collected, is represented by the posterior probability density function (pdf) defined via the Bayes’ Theorem:

(5) $$p(x|y)=\frac{p(y|x)p(x)}{p(y)},$$

where p(y|x) denotes the likelihood function, p(x) is the prior pdf and p(y) is the marginal distribution of y which is in general unknown and not computable.

#### 3.1.1 Bayesian estimators

In this section, x and y are realizations of a random variable X (with a pdf p(x)) and a random realization of Y respectively. Given a cost function $C:X\times X\to R^{+}$, a Bayesian estimator is defined as the minimizer of expected risk Ep[C(X,x^(Y))] wrt the joint distribution p(x,y) of the pair (X,Y). Several Bayesian estimators can be derived based on the choice of the cost function *C*.

  **MAP estimator** The most conventional choice is C(x,x^)=1-δ(x,x^) where δ is the Kronecker symbol. The corresponding Maximum A Posteriori estimator, defined as

(6) x^MAP=argmaxp(x|y)=argminx{-logp(y|x)-logp(x)},

selects the most likely image x, that is the solution corresponding to the mode of the posterior distribution p(x|y).

Furthermore, if we assume that the prior and likelihood functions are represented by Gibbs functions, the posterior distribution has the following form

(7) $$p(x|y)=\frac{1}{Z}\exp\left(-\frac{U(x,y)}{\tau }\right)$$

where *Z* is normalizing factor, U(x,y)=D(x,y)+ϕ(x) is an energy functional composed of a data-fidelity term D(x,y) and a prior term $\phi \left(x\right),$ and τ can be interpreted as a “temperature” or scale parameter. The prior generally encourages piecewise smoothness (TV) or sparsity of x. Hence, the MAP formulation is equivalent to the popular variational problem which amounts to computing the unique image x that minimizes the following criterion:

(8) x^=argminxD(x,y)+ϕ(x).

Typically $\phi (x)=\|\nabla x{\|}_{1}$ (∇· is the gradient operator) is the total variation regularizer and serves to smooth the image x while preserving image discontinuities.

  **MMSE estimator** Another well-known Bayesian estimator can be derived if we consider the quadratic cost function C(x,x^)=‖x-x^‖2. The Minimum Mean Square Error (MMSE) estimator is the posterior expectation (or conditional mean) defined as:

(9) x^MMSE=E[X|Y=y]=∫Xxp(x|y)dx.

If the posterior is modeled as Gibbs distribution, we get:

(10) x^MMSE=∫Xexp-U(x,y)τxdx∫Xexp-U(x,y)τdx.

In our case, the MMSE estimator is typically intractable since the underlying integral involves several thousands of variables (typically *n* is the number of pixels in the image). The MMSE estimator cannot be computed in a closed form, and numerical approximations are typically required. In high-dimensional space, a common approach consists in approximating the integral by using Monte Carlo (MC) simulation techniques (Robert and Casella, 2004) as explained in the next section.

However, we draw the readers’ attention to the fact that it has been shown that the MMSE estimator has connections with the variational optimization problem in the case of an image corrupted by white Gaussian noise:

(11) x^MMSE=argminx‖y-x‖2+ψ(x),

where the function ψ(x) can be seen as a pseudo-prior which differs from the prior distribution p(x)∝exp(-ϕ(x)). Nevertheless, except in the case of a explicit and dedicated prior discussed in (Protter et al., 2010, Gribonval, 2011, Louchet and Moisan, 2013, Kazerouni et al., 2013), it is not possible to derive an analytical form of ψ(x) from $\phi \left(x\right),$ especially if the data-fidelity term is not quadratic. Accordingly, the most practical way to compute a MMSE estimator in the case of complex data-fidelity terms and prior terms is to applying the MCMC approach.

#### 3.1.2 Monte-Carlo integration

Let us consider *T* independent and identically distributed (i.i.d.) samples $x^{\left(1\right)},\cdots x^{\left(T\right)}$ drawn from a target pdf π(x)≔p(y|x)p(x). A consistent estimator can be computed as

(12) x¯T≔1T∑t=1Tx(t)⟶px^MMSE,

i.e. the empirical mean of samples converges in probability to x^MMSE due to the weak law of large numbers. Formally, for any positive number ∊>0, we have

(13) limT→+∞Prx¯T-x^MMSE>∊=0.

The Monte-Carlo estimator is unbiased Eπ[x¯T]=x^MMSE and converges provided that the samples $x^{\left(t\right)}$ are i.i.d.. In that case, the variance of ${\overset{¯}{x}}_{T}$ defined as $\mathrm{Var}[{\overset{¯}{x}}_{T}]=\upsilon^{2}/T$ (where $\upsilon^{2}=\mathrm{Var}[X]$) decreases with the number of samples, and ${\overset{¯}{x}}_{T}$ is Gaussian distributed due to the central limit theorem: x¯T~N(x^MMSE,υ2/T) when T→+∞.

A central question is then to draw a series of i.i.d. samples. The most conventional approach in high-dimensional space is to consider Markov chain Monte Carlo (MCMC) algorithms (Gilks et al., 1995, Robert and Casella, 2004, Liang et al., 2010). In the sequel, we focus on a few important components of the MCMC machinery and we ss the convergence properties of the Metropolis-Hastings algorithm used in our approach to generate a Markov chain with a target stationary distribution π.

**Simulating independent samples and fusion of multiple chains** Drawing independent samples from the target pdf π(x) cannot be directly applied. A MCMC procedure is able to simulate an ergodic and stationary Markov chain given a target pdf π(x) and a starting state $x^{\left(0\right)}$. The set of samples $x^{\left(0\right)}\to \cdots \to x^{\left(T\right)}$ are generally correlated samples, but it has been established that Monte-Carlo estimator is consistent as T→+∞. In (Louchet et al., 2008, Louchet and Moisan, 2013), the authors also studied the behavior of the expected approximation error

(14) E[‖x~T-x^MMSE‖2]=E[‖x~T-E[x~T]‖2]+‖E[x~T]-x^MMSE‖2

decomposed into the sum of the trace of the covariance matrix (or span) and the squared bias which entails the loss of efficiency of the sampling procedure.

In practice, a MCMC method will provide better performance than another MCMC method if the samples present less correlation. On the contrary, it is required to generate many samples to reduce the variance of the estimator.

Finally, if we consider another Markov chain x′ defined as x, it is established that (Louchet et al., 2008, Louchet and Moisan, 2013): $E[∥{\tilde{x}}_{T}-{\tilde{x}}_{T}^{\prime }{∥}^{2}]=2\;E[∥{\tilde{x}}_{T}-E[{\tilde{x}}_{T}]{∥}^{2}]$. It follows that the average of the two chains has a smaller span than the span of each independent chain while keeping the same bias, i.e..

Ex~T+x~′T2-x^MMSE2⩽12E∥x~T-E[x~T]∥2+∥E[x~T]-x^MMSE∥2.

This suggests that averaging multiple independent Markov chains should provide better estimators.

**Burn-in phase** Another consequence of the correlation is the burn-in period that the chain requires before converging to the invariant target pdf π. In general, the initial $T_{b}$ samples are discarded and not included in the computation of the estimator (Robert and Casella, 2004):

(15) $${\overset{\sim }{x}}_{T}≔\frac{1}{T-T_{b}}\overset{T}{\underset{t=T_{b}}{\sum }}x^{\left(t\right)}\text{.}$$

However, the length $T_{b}$ of the burn-in period cannot be easily predicted even if a few studies in the literature focused on that problem (Gelman and Rubbin, 1992, Brooks and Gelman, 1998).

**Metropolis-Hastings algorithm** The most popular and widely applied MCMC algorithm is based on the Metropolis-Hastings procedure (Metropolis et al., 1953, Hastings, 1970) described below. The MH algorithm involves the definition of the proposal density q(z|x),x,z∈X to move from the state x to state z, and the acceptance probability 0⩽a(x,z)⩽1. The transition probability is then defined as: p(z|x)=q(z|x)a(z,x). In the MH procedure, a sample z is drawn from the proposal distribution and then a test is applied to accept the transition from the state x to the state z or not. If the transition is not accepted, the chain remains in the same state as before:

  ***The Metropolis-Hastings algorithm***

1. Set an initial state $x^{\left(0\right)}$.
2. **For** t=1,⋯,T **do**
  (a) Draw a sample $z\sim q(x|x^{\left(t-1\right)})$.
  (b) Compute the acceptance probability
    $$a(x^{\left(t-1\right)},z)=\min\left[1,\frac{\pi (z)q(x^{\left(t-1\right)}|z)}{\pi (x^{\left(t-1\right)})q(z|x^{\left(t-1\right)})}\right]\text{.}$$
  (c) Draw α from a uniform distribution: α~U(0,1).
  (d) **If** $\alpha ⩽a(x^{\left(t-1\right)},z)$ **then** $x^{\left(t\right)}=z$,
    **else**                         $x^{\left(t\right)}=x^{\left(t-1\right)}$.
    **end if**
  **end for**

The MH algorithm returns a set of $T_{b}-T$ correlated samples if we discard the $T_{b}$ first samples. Under some mild regularity conditions, it has been established that the pdf of $x^{\left(t\right)}$ converges to the target pdf π when *t* increases (Robert and Casella, 2004). In general, the MH algorithm satisfies the so-called detailed balance condition:

(x,z)∈X×X,p(x|z)π(z)=p(z|x)π(x),

(the chain is reversible) which is a sufficient condition to guarantee that the chain is ergodic and has π as stationary distribution (Robert and Casella, 2004). Note that reversibility of the chain is not a necessary condition; recent studies experimentally show that non-reversible Markov chains may provide better convergence, i.e. the number *T* of samples can be lower than the reversible chains (Neal, 2004, Bierkens, 2015).

In practice, the proposal density and the acceptance probability can be modified in order to improve the performance of the algorithm, and always ensuring the ergodicity of the chain. Actually, the proposal pdf *q* should be chosen as close as possible to the target pdf π. In what follows, we mainly focus on the specification of the proposal densities to improve convergence.

**Choices of the proposal density** There is a large flexibility in the choice of proposal function and it is a challenge to find a proposal function that is able to use the data efficiently in order to obtain satisfactory convergence. Below, we ss four possible proposals.

• Assume that the proposal satisfies the equality q(z|x)=q(x|z) (e.g. uniform distribution), then the acceptance probability is simplified since a sample z having a higher value π(z) is always accepted, whereas the samples with smaller values π(z)<π(x) are accepted with a probability lower than 1.
• The proposal pdf has the following form: q(z|x)=q(z-x). This means that the new state is explicitly randomly found in the neighborhood of the current state x. This proposal is then viewed as random walk and enables to progressively explore the large space of possible states. Nevertheless, the random walk MH algorithm (1953) tends to stay in the same state for a long period but the chain has not converged.
• When the target density is differentiable, the proposal can be generated in accordance with an approximation of the Langevin diffusion process (Girolami and Calderhead, 2011): $z\sim N(x+\frac{\delta }{2}\nabla \log\pi (x),\delta )$ for a given small value δ.
• The idea of adaptive MH algorithm consists in updating the proposal distribution by using all the information collected so far about the target distribution (Holden et al., 2009). First, it has been suggested to model the proposal density as a Gaussian distribution centered on the current state with a covariance computed from a fixed finite number of previous states. Given the whole history $\left(x^{\left(0\right)},x^{\left(1\right)},\cdots ,x^{\left(t-1\right)}\right)$, the new state z is obtained from $q(z|x^{\left(0\right)},x^{\left(1\right)},\cdots ,x^{\left(t-1\right)})$ assumed to be symmetric.
• If the proposal pdf q(z|x)=q(x) does not depend on the state x, the acceptance probability is defined as
  $$a(x,z)=\min\left[1,\frac{\pi (z)q(x)}{\pi (x)q(z)}\right]\text{.}$$
  The independent Metropolis-Hastings algorithm is an efficient sampling algorithm only if *q* is reasonably close to π. An attractive property of independent proposals is their ability to make large jumps while keeping the acceptance rate high. Consequently, the autocorrelation of the chain decreases rapidly.

Note that more sophisticated proposal rules are generally recommended to address high-dimensional problems (Girolami and Calderhead, 2011). For instance, a two-step optimization approach is typically appropriate for sampling Gaussian distributions (Papandreou and Yuille, 2010, Gilavert et al., 2015). Another sophisticated approach is based on data augmentation and the adding of auxiliary variables to improve convergence speed if the samples are correlated (Marnissi et al., 2018).

In summary, the convergence of the chain depends on the specification of the proposal density but it is also established that the ideal proposal pdf must as close to possible to the target pdf. In that case, the MH procedure generates a sequence of states with low correlations and converges faster. In our approach described in the next section, we investigated a stochastic scheme to generate samples with low correlations in the context of MW restoration.

## 4 Our MH algorithm for missing wedge restoration

### 4.1 Gibbs energy modeling

Let us consider the following image model

(16) $$y=D_{W}(x),$$

where $y,x\in R^{n}$, and $D_{W}(.)$ is a degradation operator setting to zero the Fourier coefficients belonging to the MW support *W* assumed to be known. Our objective is to compute a MMSE estimator defined as

(17) x^MMSE=∫Xp(x|y)xdx∫Xp(x|y)dx=∫Xp(y|x)p(x)xdx∫Xp(y|x)p(x)dx

by using a dedicated MH algorithm, where p(x) is the prior used to encourage the solution to be positive and piecewise smooth. In our modeling, the likelihood function is composed of two terms: i/ we impose that the Fourier coefficients of the reconstructed image are very similar to the known coefficients of the corrupted image y, i.e. $|Fx\left[k\right]-Fy\left[k\right]|<\delta ;$ ii/we consider a data fidelity term defined as the $L_{2}$ norm (alternative data fidelity terms will be considered in our experiments) between the corrupted image and the restored image degraded by the operator $D_{W}$, i.e..

(18) $$D(x,y)=\underset{s\in S}{\sum }{\left(y(s)-\underset{k\in S}{\sum }e^{2i\pi k\cdot s}Fx[k]\right)}^{2}\text{.}$$

It follows that the posterior is defined as ($1_{A}(z)=1$ if z belongs to the set A and zero otherwise)

(19) $$p(x|y)\;\propto \;\underset{\mathrm{likelihood}}{\underset{︸}{\exp\left(-\frac{D(x,y)}{\beta }\right)1_{A_{\delta ,y}}(x)}}\;\underset{\mathrm{prior}}{\underset{︸}{1_{A_{\lambda }^{\prime }}(\nabla x)\;1_{X_{+}}(x)}},$$

such that $A_{\delta ,y}(x)=\{x\in X:\forall k\in \overset{‾}{W}\;\;|Fx[k]-Fy[k]|<\delta \}$ and $A_{\lambda }^{\prime }(\nabla x)=\{x\in X:\|\nabla x{\|}_{1}⩽\lambda \}$ where $\left\|\nabla x{\|}_{1}=\sum_{s\in S}|\nabla x(s)\right|$ is the discrete Total Variation (Rudin et al., 1992). Here, $X_{+}$ denotes the set of positive solutions, that is the set of images for which $x\left(s\right)⩾0,\forall s\in S.$ As mentioned in Section 3.1, β can be interpreted in (19) as a “temperature” parameter. In Eq. (19), the prior term imposes positivity and regularity of the solution. The TV norm of the restored image is then assumed to be lower than a prescribed threshold λ. The likelihood is based on reconstruction error (18) and adequacy of frequency in the SR region $\overset{‾}{W}$.

Consequently, the MMSE estimator can reformulated as

(20) x^MMSE=∫Γexp-D(x,y)βxdx∫Γexp-D(x,y)βdx,

where the set Γ of admissible solutions is defined as

$$\Gamma =\{x\in X:\forall k\in \overset{‾}{W}\;\;|Fx[k]-Fy[k]|<\delta ,\;\forall s\in S,\;x(s)⩾0,\;and\;\|\nabla x{\|}_{1}<\lambda \}\text{.}$$

The performance of MMSE (20) depends on the pre-specified thresholds λ and δ. In practice, these values do not need to be accurately adjusted in practice as ssed below. Meanwhile, because of the high dimensionality of the problem, we need an efficient MH algorithm to compute MMSE.

### 4.2 Implementation of the MH algorithm

The efficiency of a MH algorithm depends on the choice of the proposal distribution $q(.|x^{\left(t-1\right)})$. In practice, the proposal generator leads to correlated samples $x^{\left(t-1\right)}$ induced by the two following factors: i/ by construction, the newly proposed state $x^{\left(t\right)}\sim q(.|x^{\left(t-1\right)})$ is generated from the current state; ii/ the new state $x^{\left(t\right)}=x^{\left(t-1\right)}$ when the proposed move has been rejected. Note that this correlation is not known in advance but can be empirically estimated and updated from the previous samples (Holden et al., 2009). To achieve good performance, a well-chosen proposal distribution both allows significant changes between the subsequent states with a high probability of acceptance (see Section 4.2.4). Unfortunately these requirements cannot be satisfied easily in practice. If we choose a proposal distribution with small moves, the probability of acceptance will be high, however the resulting chain will be highly correlated, as $x^{\left(t\right)}$ changes only very slowly. In return, if we choose a proposal distribution with large moves, the probability of acceptance will be rather low. Accordingly, we investigated an original strategy to generate a sequence of moves with a probability of acceptation in the range of a∈[0.25-0.6]. In theoretical studies, it has been shown that the optimal probability of acceptance $a^{☆}=0.234$ (Roberts et al., 1997) whereas $a^{☆}=0.574$ in Roberts and Rosenthal (2001).

Given an initial state $x^{\left(0\right)}\in \Gamma$, we explore the neighborhood of the current value set $x^{\left(t\right)}$ of the chain. Our proposal distribution *q*, which enables to potentially move from x∈Γ to $x^{\prime }\in \Gamma$ is chosen as (also see Fig. 2):

(21) $$x^{\prime }=D_{\lambda }(P_{W}(x+\varepsilon )),$$

where $\varepsilon \sim N(0,I_{n\times n}\sigma_{\varepsilon }^{2})$ is a white Gaussian noise, $I_{n\times n}$ is the *n*-dimensional identity matrix, $P_{W}$ is a projection operator that impose that the $\forall k\in \overset{‾}{W},\;\;F(x+\varepsilon )[k]=Fy[k]$ and $D_{\lambda }$ is denoising operator that ensures that the total variation of the denoised image is lower than λ. Consequently, the distribution of increments $x^{\prime }-x$ is not parametric due to the nonlinear operators involved in (21). In the sequel, we only assume that this non-parametric distribution *q* is approximately symmetric. Even though visualization of the empirical proposal density is not possible, we suggest that (21) tends to produces similar samples (denoised images) concentrated around some empirical mean belonging to Γ, with a few moves quite far away from this mean.

Our simulator can be viewed as a random walk in a high-dimensional space, where all the pixels of the images are modified at once. Note that in (Louchet et al., 2008, Louchet and Moisan, 2013), only one pixel is modified at each iteration to compute the TV-LSE estimator. Our approach can be seen also a blockwise MH sampling procedure but only one block corresponding to the whole image, is considered in procedure. In our experiments, we observed a high acceptance rate, suggesting that the new sample is not far from the previous one (i.e. $∥x^{\left(t\right)}-x^{\left(t-1\right)}∥$ is small). To our knowledge, this is the first time that such a proposal, is used in the context of image restoration and inverse problems. Our MH algorithm for MW restoration is then as follows (see Fig. 2):

  **The Missing Wedge Restoration (MWR) algorithm**

Set an initial state $x^{\left(0\right)}\in \Gamma$.

**For** t=1,⋯,T **do**

1. Generate a new state z with the a three-step approach:
  • **Perturbation**: $x^{\left(t-1\right)}+\varepsilon ,\varepsilon \sim N(0,I_{n\times n}\sigma_{\varepsilon }^{2})$.
  • **Projection:** of $P_{W}(x^{\left(t-1\right)}+\varepsilon )$ onto the subspace of images having the same observed frequencies as y if $k\in \overset{‾}{W}$.
  • **Denoising** of $P_{W}(x^{\left(t-1\right)}+\varepsilon )$ to get an image with a small $||\nabla x|{|}_{1}$ and set $z=D_{\lambda }(P_{W}(x^{\left(t-1\right)}+\varepsilon ))$.
2. Compute the acceptance probability
  $$a(x^{\left(t-1\right)},z)=\min\left[1,\exp\left(\frac{D(x^{\left(t-1\right)},y)-D(z,y)}{\beta }\right)\right]\text{.}$$
3. Draw α from a uniform distribution: α~U(0,1)
4. **If** $\alpha ⩽a(x^{\left(t-1\right)},z)$ **then** $x^{\left(t\right)}=z$
  **else**                         $x^{\left(t\right)}=x^{\left(t-1\right)}$.
  **end if**

**end for**

### 4.3 Setting of parameters

In the end, the computational method is governed actually by three parameters: the number of iterations *T*, the noise variance $\sigma_{∊}^{2}$ and the scaling parameter β. At each iteration of the procedure, we consider that any state-of-the-art algorithm produces a satisfyingly smooth image, that is an image for which the total variation is lower than λ. In practice, it is not required to accurately set this parameter λ. At each iteration *t*, the denoising algorithm removes the perturbation noise. The parameter β affects the acceptance rate of the evaluation step. The higher the value of β, the higher the acceptance rate. For a high enough β value, all proposed samples are accepted and we fall back on the original method (Maggioni et al., 2013).

Unlike Maggioni et al. (2013), we propose a statistical physics and energy minimization framework for MW restoration. In (Maggioni et al., 2013), all candidate samples are accepted at each iteration and used to compute an aggregated estimator. It is worth noting that in (Maggioni et al., 2013), the standard deviation $\sigma_{∊}$ decreases through iterations, and the final estimate is obtained by weighting the samples $x^{\left(t\right)}$ with weights equal to $1/\sigma_{∊}$ updated at each iteration. Hence, this gives more importance to the last samples. In our case, $\sigma_{∊}$ is constant through iterations, and the weights results naturally from the MCMC sampling which selects the most appropriate generated samples. The most frequent accepted samples have higher weights in the computation of the Monte-Carlo estimator (Eq. 12).

Similar to (Maggioni et al., 2013), we used BM4D as denoising operator in MWR. Actually BM4D uses two denoising steps: Step #1 is a hard thresholding performed in the discrete cosine transform domain; Step #2 is Wiener filtering. To save computing time (about factor 2), we focused on Step #1 in our iterative MWR algorithm. Additional experiments on real cryo-ET data (see Fig. 13) also confirmed that the denoising results were worse after Step #2.

Finally, we add a “periodic plus smooth image decomposition” (Moisan, 2011) operation prior to Fourier transforms, in order to reduce artifacts originating from image borders. Indeed, we noticed that a cross-structure tends to emerge in the restored MW and gets amplified through iterations. This structure is a well-known spectral artifact of the Fourier transform, resulting from the false assumption that the images are periodic signals, when in reality the images rarely have similar opposite borders. Applying this decomposition allows to reduce the cross structure and solves the problem.

## 5 Experimental results

The MW restoration method has been evaluated experimentally on synthetic noisy data by varying the parameters and the components of the MH algorithm. Furthermore, we demonstrate the potential of the method on real cryo-ET data.

### 5.1 Results on synthetic data

In this section, we justify the choice of algorithm parameters, then evaluate robustness to noise, and finally compare our approach to a few other competitive methods. We consider an artificial dataset (Dataset A) which consists of a density map of the 20S proteasome corrupted by additive white Gaussian noise and by applying an artificial MW process, which amounts to setting to zero the Fourier coefficients within an artificial wedge shaped mask. Given the ground-truth x, we use two similarity measures for quantitatively evaluating the restoration results x^: the peak signal-to-noise ratio (PSNR) and the constrained correlation coefficient (CCC), defined as

(22) PSNR(x,x^)=maxs∈Sx(s)1n∑s∈Sx(s)-x^(s)2,

(23) CCC(x,x^)=∑k∈WFx[k]Fx^[k]∗∑k∈WFx[k]2∑k∈WFx^[k]2.

The PSNR is a common score in image denoising, and is well adapted to estimate the global quality of processed images. CCC is a score used in cryo-ET for sub-tomogram averaging (Förster and Hegerl, 2007). This score is very similar to the Pearson’s correlation coefficient measured in Fourier domain. Note that only a constrained region of the Fourier domain is considered for CCC computation. In our work, we use CCC to quantify the quality of retrieved Fourier coefficients located in the region *W*.

#### 5.1.1 Analysis of performance of denoising algorithms

**Fig. 3:**
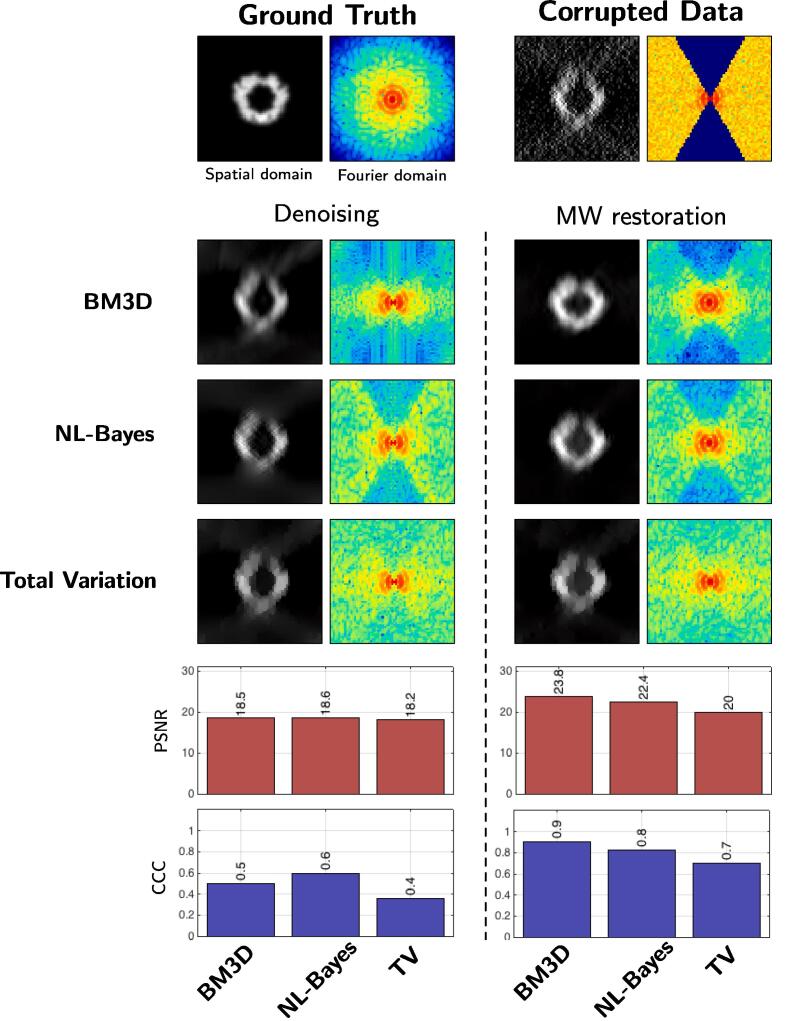
Dataset A (2D): comparison of denoising algorithms. First, we compare conventional denoisers (left column) to our MH method (right column). Second, we compare the results obtained by applying three different 2D denoising algorithms: BM3D, NL-Bayes, and ROF denoising. On the bottom, we evaluate the performance in terms of PSNR and CCC values. It turns out that our MH method performs better than conventional denoisers in all situations.

First, we study the influence of several denoising algorithms embedded in our MCMC framework. We focus on three 2D methods: BM3D (Dabov et al., 2007), non-local Bayes (NL-Bayes) (Lebrun et al., 2013), total variation (TV) (Rudin et al., 1992). Since it was not possible to extend all denoisers in 3D, we applied the method on a 2D slice of the 3D volume for assessment. As shown in Fig. 3, the best results have been obtained by using BM3D, both in terms of PSNR and visual quality. Nevertheless, NL-Bayes produced very similar results. TV denoising produced noticeable worse results. It turns out that the performance strongly depends on the ability of the denoising algorithm to remove the Gaussian noise. This experiment confirms that our MCMC procedure achieves better results than traditional denoising algorithms. In addition, the best results are obtained with the BM3D and NL-Bayes algorithms embedded in our MH algorithm. This is consistent with the literature in image denoising. Finally, we confirm that any image denoising algorithm (we also tested NL-means (Buades et al., 2005) PEWA (Kervrann, 2014), OWE (Jin et al., 2017)) allow us to produce a restored image with less MW artifacts. Because faster and very performant in terms of PSNR values, we used BM4D (Maggioni et al., 2013) which a 3D extension of BM3D, for processing 3D cryo-ET data.

#### 5.1.2 Acceptance rate of MH algorithm

**Fig. 4:**
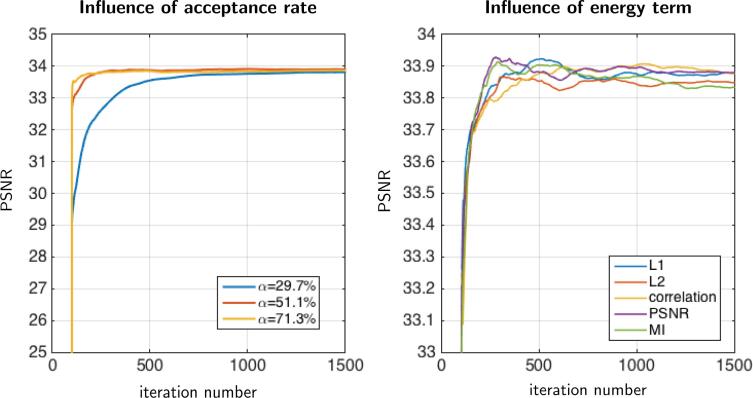
Dataset A: This figure shows the impact of algorithm parameters. We illustrate these effects in terms of PSNR through iterations. We do not show images because obtained results are visually similar. On the left, the influence of parameter β controlling the acceptance rate of the MH sampling, is shown: $\beta =1.5\times 10^{-5}$ (blue), $\beta =2.0\times 10^{-5}$ (red), $\beta =4.0\times 10^{-5}$ (yellow). Clearly, the choice of β affects the convergence speed, however in all cases our method converges to the same result. On the right, the influence of the data fidelity term is presented. We compare the $L_{1}$(24) and $L_{2}$ norms (25), the correlation coefficient (26), the PSNR (22), and the mutual information (27). The results are very similar (maximum error of 0.1 dB between restored images). (For interpretation of the references to colour in this figure legend, the reader is referred to the web version of this article.)

In this section, we evaluate the sensitivity of the parameter β controlling the acceptance rate of the MH procedure (combined with BM4D). In Fig. 4(left), it is confirmed that the acceptance rate affects the convergence speed. Nevertheless, whatever the parameter β chosen in the range $[1.5-4.0]\times 10^{-5}$, the algorithm provides solutions with a similar PSNR value close to 34 dB. Note that we got similar reconstructed images by uniformly aggregating all the samples or by aggregating samples with weights equal to the exponential form of the data fidelity term.

In theory, the recommended acceptation rate is about 0.234 (Breyer and Roberts, 2000) in the MH algorithm if we consider a Gaussian proposal distribution. In our case, we get faster convergence since the maximum acceptation rate is about 70%, suggesting that the set of proposed samples are relevant.

In what follows, we set $\beta =4.0\times 10^{-5}$ since it provides faster convergence as shown in Fig. 4(left).

#### 5.1.3 Data-fidelity terms

We have tested several data-fidelity terms D(x,y), corresponding to PSNR (see (22)), $L_{1}$ and $L_{2}$ norms defined as

(24) $$D_{L_{1}}(x,y)=\underset{s\in S}{\sum }|x(s)-y(s)|,$$

(25) $$D_{L_{2}}(x,y)=\underset{s\in S}{\sum }{\left(x(s)-y(s)\right)}^{2},$$

and the Pearson’s correlation coefficient (CC):

(26) $$D_{\mathrm{CC}}(x,y)=\frac{\underset{s\in S}{\sum }(x(s)-\mu_{x})(y(s)-\mu_{y})}{\sqrt{\underset{s\in S}{\sum }{\left(x(s)-\mu_{x}\right)}^{2}}\sqrt{\underset{s\in S}{\sum }{\left(y(s)-\mu_{y}\right)}^{2}}},$$

where $\mu_{x}$ and $\mu_{y}$ are the means of x and y, respectively. We have also evaluated a data-fidelity term based and the mutual information (MI):

(27) $$D_{\mathrm{MI}}(x,y)=\underset{i,j}{\sum }p_{\mathrm{xy}}(i,j)\log\frac{p_{\mathrm{xy}}(i,j)}{p_{x}(i)p_{y}(j)},$$

where $p_{\mathrm{xy}}(i,j)$ is the joint probability function of x and y with intensity bins *i* and *j*, and $p_{x}(i)$ and $p_{y}(j)$ denote the marginal probability distribution functions of x and y, respectively. In our implementation, we approximate the probability functions by histograms of pixel values.

It turns out that the resulting images are very similar for all these data-fidelity terms (see Fig. 4(right)). We observed a maximum error of 0.1 db between the final restored images. In the sequel, we decided to focus on the $D_{L_{2}}$ data-fidelity term to evaluate the components of the MH algorithm.

#### 5.1.4 Spectral analysis of MW

**Fig. 5:**
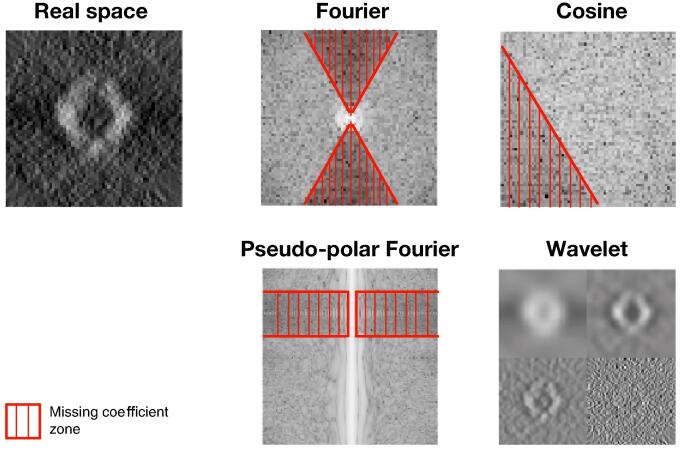
The missing wedge shape (in red) for different transforms: Fourier transform, cosine transform, and pseudo-polar Fourier transform. Note that the missing wedge is not apparent in all transforms, as is illustrated with the wavelet transform.

**Fig. 6:**
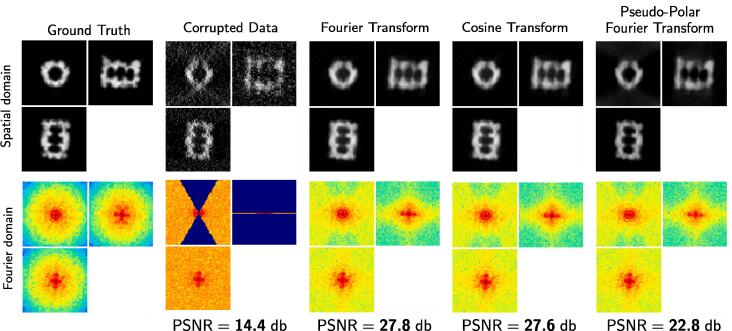
Dataset A (3D): influence of the transform type for MW restoration. From left to right: the ground truth (reference for measuring the PSNR), the corrupted image (used as input for the method), and the processed images using the Fourier transform, cosine transform and pseudo-polar Fourier transform. The best result is obtained by using the Fourier transform.

The MW has different shapes if we consider different reciprocal spaces as illustrated in Fig. 5. In our framework, any spectral transform, provided that the transform allows us to decompose the spectral domain into connected components, that is two regions corresponding to non-zero and zero coefficients. The wavelet transform is typically not appropriate as shown in Fig. 5. The MW region should be as small as possible to make restoration successful. Accordingly, we investigated several spectral transforms to improve image restoration with our approach (see Fig. 6). The best result is achieved with the discrete fast Fourier transform (FFT), followed very closely by the discrete cosine transform (DCT). The pseudo-polar fast Fourier transform (PP-FFT), already considered in cryo-ET (Miao et al., 2005), achieves a worse result, both visually and in terms of PSNR values. Finally, it turns out that the performance of our method is not impacted by the transform type, but is actually influenced by the potential precision of the inverse transform. When evaluating the implementations of the considered three transforms, the resulting mean squared errors are in the range of $10^{-34}$ for DFT, $10^{-32}$ for DCT and $10^{-12}$ for PP-DFT. These errors perfectly correlate with the performance given in Fig. 6. Therefore, similar results could be achieved with another transform, provided that the inverse transform is precise enough.

#### 5.1.5 Comparing MAP and MMSE estimators

Both the MAP and MMSE have been used considered for solving image restoration problems. For instance, in (Louchet et al., 2008, Louchet and Moisan, 2013), the authors compared TV-MAP and TV-LSE and consider the TV norm as a prior to encourage piece-wise constant images. TV-MAP is more appropriate for restoring piecewise constant images (e.g. cartoons, shepp-Logan phantom), but washes out textures present in natural images and in microscopy images. To reduce stair-casing artifacts produced by TV-MAP, Louchet and Moisan (2013) proposed the TV-LSE estimator to favor smooth transitions between contrasted regions instead of sharp ones.

**Fig. 7:**
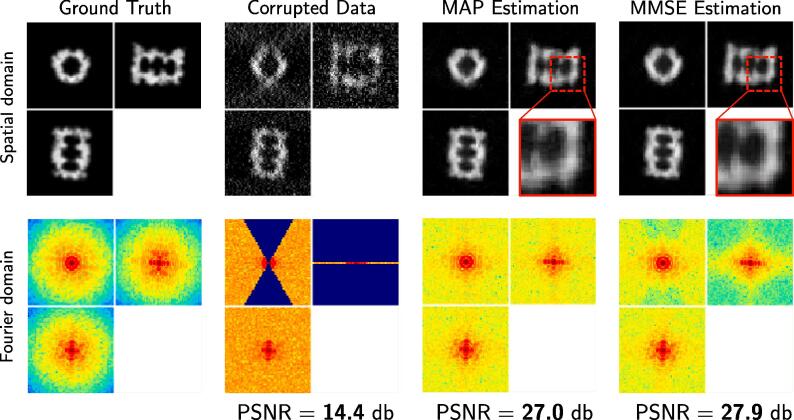
Dataset A (3D): comparison of MAP and MMSE estimators. From left to right: the ground truth (reference for PSNR), the corrupted image, the MAP and MMSE estimators computed with our MCMC procedure. As can be observed in the zoomed in regions (the red frames), the MMSE estimator is less noisy than the MAP estimate, with a higher PSNR value.

In our modeling framework, we do not directly use TV as in (Louchet et al., 2008, Louchet and Moisan, 2013). The TV constraint is actually used to a priori discard irrelevant samples during the proposal step in the MCMC sampling procedure. Nevertheless, we can compare the performance MMSE estimator to the MAP estimator corresponding to the most frequent sample in the MCMC sequence. At first glance, both estimates look visually similar, even though the MAP estimate produces more background noise (see Fig. 7). The difference is more noticeable in the spectral domain: the MW of the MAP estimate contains higher amplitudes in the high frequencies. However, this does not mean that the MW restoration is of better quality. Indeed, according to the PSNR values, MMSE is closer to the ground-truth than any generated sample. Therefore the higher MW amplitudes in the MAP estimate most likely carry noise rather than meaningful information.

#### 5.1.6 Robustness to noise, comparison to BM4D (Maggioni et al., 2013)

**Fig. 8:**
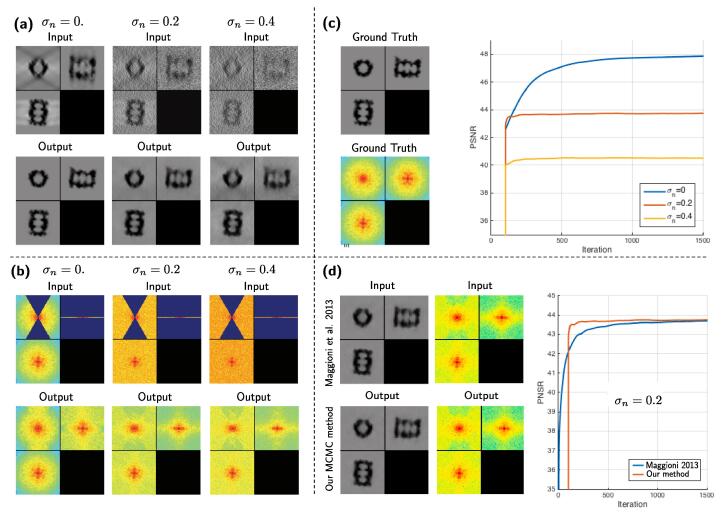
Simulated data of the 20S proteasome, for varying amounts of noise (dataset A). All images depict ortho-slices of 3D volumes. The volume size is 64×64×64 voxels. For (a) and (b), top row: method inputs, bottom row: method outputs. Results are shown in spatial domain (a) and spectral domain (b). In (c) can be observed the ground-truth and the increase of the PSNR values over iterations. In (d) we compare our method to the original method (Maggioni et al., 2013).

From the results on dataset A (see Fig. 8(a)), it can be seen how well our method works in the absence of noise ($\sigma_{n}=0$): a quasi perfect image recovery has been achieved, despite the complexity of the object. Increasing the amount of noise deteriorates the performance, but as it can be observed for $\sigma_{n}=0.2$, the result is still satisfying. For high amounts of noise ($\sigma_{n}=0.4$), the object contrast is still greatly enhanced but the MW artifacts could not be completely removed. In Fourier domain (Fig. 8(b)), the MW has been filled up completely when $\sigma_{n}=0,$ whereas for an increasing amount of noise the MW reconstruction is increasingly restrained to the low frequencies. This is because high frequency components of a signal are more affected by noise, which makes them more difficult to recover. In Fig. 8(c), the evolution of the PSNR over time shows that the method converges to a stable solution.

In Fig. 8(d), we compare our MH method to the method proposed by Maggioni et al. (2013). Both methods produce visually identical results in spatial domain, as well as in the spectral domain, as confirmed by the final PSNR values. However, the difference lies in the convergence speed: our method takes about half as long as the original method (Maggioni et al., 2013). Even though the synthetic dataset A is a simplified case of data corruption in cryo-ET, it gives a good intuition of the performance of our method.

#### 5.1.7 Comparison to other MW restoration methods

**Fig. 9:**
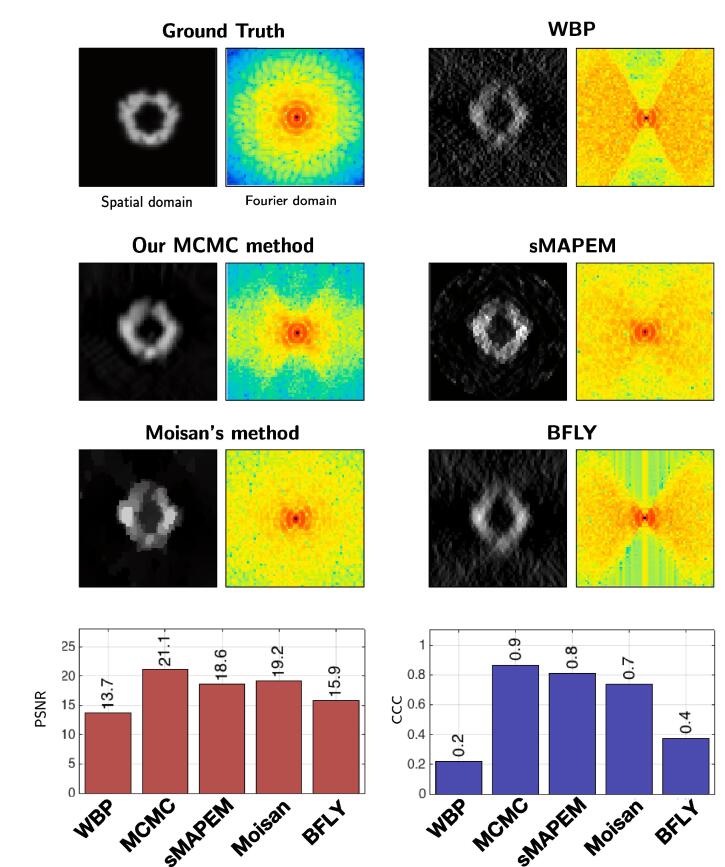
Dataset A (2D): comparing our approach to competitive methods: i/sMAPEM, a regularized tomographic reconstruction method designed to achieve isotropic resolution; ii/ the Moisan’s method designed to extrapolate missing regions in Fourier space; iii/BFLY, a filter designed to reduce MW artifacts. The sMAPEM method takes projections as input, therefore we used the same projections to produce the 2D input (via WBP) for the other methods. On the bottom we display the PSNR and CCC scores obtained for all tested methods.

We compare our results to those produced by three competing methods (see Fig. 9): sMAPEM (Paavolainen et al., 2014), BFLY (Kováčik et al., 2014), and a TV method with spectral constraints (Moisan, 2001), each adopting a different strategy to reduce MW artifacts. We implemented the Moisan’s method as follows:

**Our implementation of the** Moisan (2001)**’s** **method:**

Set an initial state $x^{\left(0\right)}\in \Gamma$.

**For** t=1,…,T **do**

1. **Projection:** $z^{\left(t\right)}=P_{W}(x^{\left(t\right)})$
2. **Denoising:** $\min_{x}∥z^{\left(t\right)}-x{∥}^{2}+\lambda \|\nabla x{\|}_{1}$

**end for**which amounts to alternatively minimizing TV (Rudin et al., 1992) and satisfying the spectral constraints. The sMAPEM algorithm (Paavolainen et al., 2014) is an iterative tomogram reconstruction procedure, designed to reduce MW artifacts and achieve isotropic resolution. In our experiments, we performed the comparison in 2D mainly because sMAPEM is not available in 3D. Our method, BFLY and the TV method operate on tomograms (2D in our case), while sMAPEM operates on projections (1D). These projections were obtained from the dataset A ground-truth, with a tilt-range of −60°–60° and a tilt increment of 2 degrees. We then added Gaussian noise ($\sigma_{n}=0.17$) to the projections. In order to make a fair comparison, the inputs should originate from these same projections. Therefore we use the weighted back-projection (WBP (Radermacher, 1992)) algorithm to produce the 2D data needed for the other methods.

As explained above, the strategy of sMAPEM differs from ours, in the sense that it takes as an input projections and gives as an output a reconstructed tomogram. One may argue that the best strategy would be the one adopted by sMAPEM, because it directly uses the projections, instead of a tomogram which is already contaminated by MW artifacts. However, as shown in Fig. 9, our method achieves a better PSNR value than sMAPEM. Visually both methods seem to approach isotropic resolution, however the result of our method is smoother, while the result of sMAPEM contains pixelated artifacts. The result of the Moisan’s method is visually satisfying. In real space, noise appears to be removed, however the result suffers from staircasing artifacts, as well known with TV denoising. In Fourier space, the entire MW appears to be restored, as opposed to our method and sMAPEM where the restoration is constrained to lower frequencies. However according to CCC values, these restored Fourier coefficient do not correlate as well with the ground truth as our method and sMAPEM. The BFLY filter aims at reducing the MW ray-artifacts by smoothing the sharp transition between the MW and the SR. The object elongation and side artifacts however remain. Unlike other competing methods, BFLY does not recover missing Fourier coefficients, it is therefore not surprising that it has the worst performance, both visually and in terms of PSNR and CCC values.

In summary, our approach outperforms competing methods both in terms of PSNR and CCC values. Visually, our approach produces a smoother image, while sMAPEM and the Moisan’s method introduce artifacts in the result. The weakest performance is achieved by BFLY, however this was expected as the other methods have much higher complexity. That being said, BFLY is the fastest method.

### 5.2 Results on experimental data

In this section, we evaluate the performance of our MH method (combined with BM4D) on real images to confirm the results obtained on artificial images.

#### 5.2.1 Experimental datasets

Three datasets (B, C and D) have been used to evaluate the performance of the method on experimental data. Dataset B is an experimental sub-tomogram containing a gold particle. Dataset C is an experimental sub-tomogram containing 80S ribosomes attached to a membrane. Dataset D is an experimental sub-tomogram depicting a region of an E. Coli bacteria, and contains unidentified macromolecules next to a membrane. Unlike data-sets B and C, the dataset D is available as single-axis and double-axis data (see Section 1).

#### 5.2.2 Criteria and method for evaluation

The evaluation differs depending on the dataset. In dataset B, the gold particle is deformed and elongated (ellipse) due to the MW artifacts. Improving the sphericity of the object is thus a good evaluation criterion. For dataset C, we measure the similarity between the central ribosome and a reference (obtained via sub-tomogram averaging). The evaluation criterion is the Fourier shell correlation (FSC), commonly used in cryo-ET (Van Heel and Schatz, 2005). In order to measure the quality of the recovered MW only, we also compute the FSC over the MW support (“constrained” FSC or cFSC). For dataset D, we have both single-axis and double-axis versions of the data. A double-axis volume has less missing Fourier coefficients than a single-axis volume. Therefore, when processing the single-axis volume, the additional Fourier coefficients of the double-axis volume can act as an experimental ground truth. We evaluate the result with the CCC score, as illustrated in Fig. 13.

#### 5.2.3 Analysis of restoration results

**Fig. 10:**
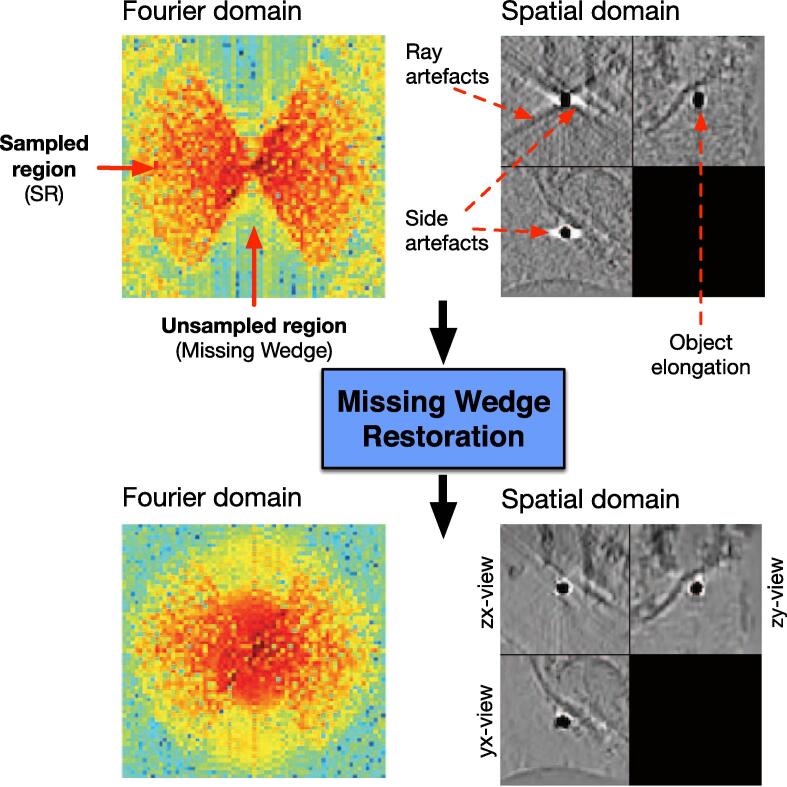
Experimental sub-tomogram (61×61×61 voxels) containing a gold particle (dataset B). The top row shows the input in the spectral and spatial domains, the bottom row shows the restored image and spectrum.

The result on dataset B shows that noise is reduced and a significant part of the MW was recovered (see Fig. 10). Even though the recovery is not complete, it is enough to reduce the MW artifacts while preserving and enhancing image details. The ray and side artifacts induced by the high contrast of the gold particle are reduced and its sphericity has been improved, bringing the image closer to the expected object shape. The result on this dataset shows that the method is able to handle experimental noise in cryo-ET.

**Fig. 11:**
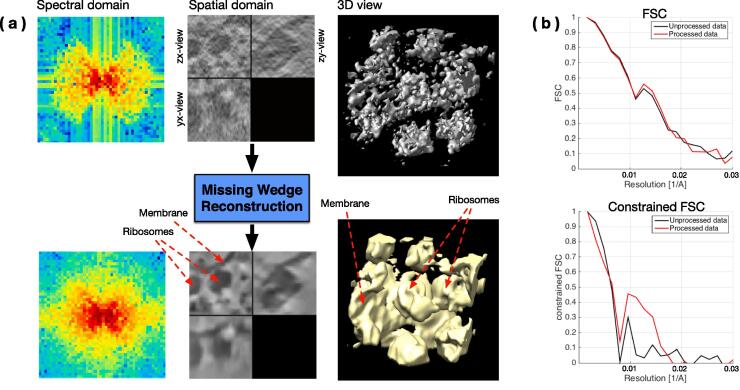
Experimental sub-tomogram (46×46×46 voxels) containing ribosomes attached to a membrane (dataset C). (a) Top row: input image in spectral domain, spatial domain and 3D view of the thresholded data. Bottom row: the same representations for the output. (b) FSC and cFSC measures of the method input (in black) and output (in red). The FSC measures overall quality, while the cFSC measures quality of recovered Fourier coefficients only (i.e. MW). All measures are wrt the same reference. (For interpretation of the references to colour in this figure legend, the reader is referred to the web version of this article.)

**Fig. 12:**
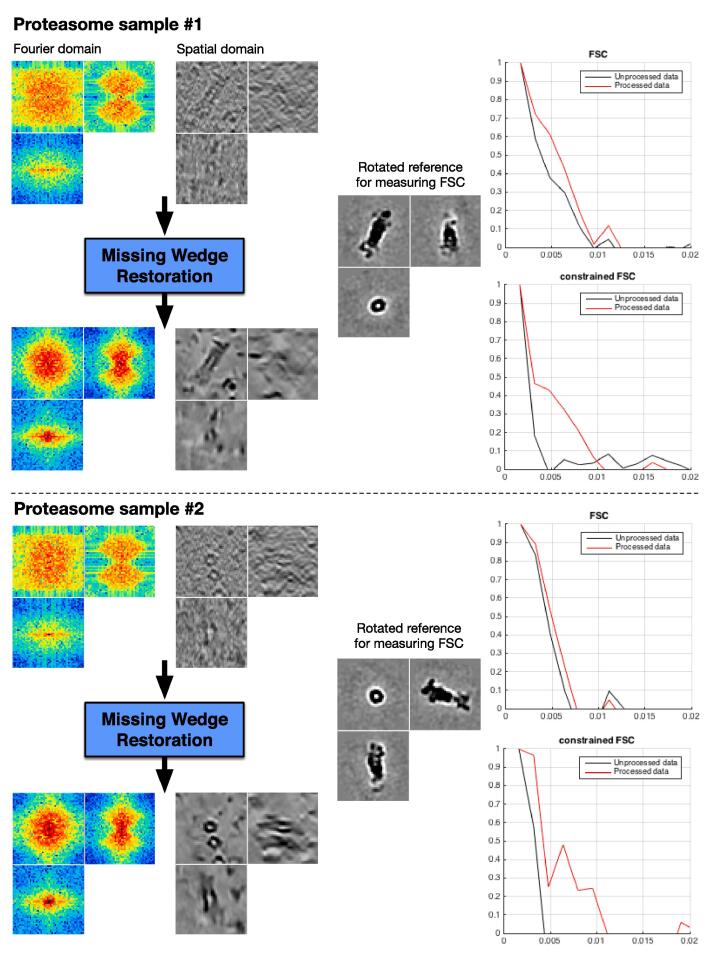
Two experimental sub-tomograms (46×46×46 voxels) containing proteasomes. Data is displayed in both Fourier and spatial domains. We evaluate the result with FSC and cFSC measures of the method input (in black) and output (in red). The FSC measures overall quality, while the cFSC measures quality of recovered Fourier coefficients only (i.e. MW). The reference has been obtained via subtomogram averaging of 2949 proteasomes. (For interpretation of the references to colour in this figure legend, the reader is referred to the web version of this article.)

The dataset C (ribosomes, Fig. 11) is more challenging because the objects have a more complex shape and less contrast, i.e. the SNR is lower. Nonetheless, the method significantly enhanced the contrast and, according to the FSC criteria, the signal has indeed been improved. Although visually it is more difficult to conclude that the MW artifacts have been affected, the Fourier spectrum shows that Fourier coefficients were recovered. As expected, the amount of recovered high frequencies is less than for dataset B, because of the lower SNR. It is now necessary to provide a proof that the recovered coefficients carry a coherent signal, therefore the cFSC has been measured. The black curve in Fig. 11 depicts the cFSC between the unprocessed image and the reference: given that the MW contains no information, the curve represents noise correlation. Consequently, everything above the black curve is signal, which is indeed the case for the processed data (red curve in Fig. 11). To illustrate how the method can improve visualization, a simple thresholding has been performed on the data (3D views in Fig. 11). While it is difficult to distinguish objects in the unprocessed data, the shape of ribosomes become clearly visible and it can be observed how they are attached to the membrane. In addition, in order to demonstrate that our procedure is not limited to ribosomes (considered as easy targets because of their good contrast), we perform the same evaluation procedure (i.e. dataset C) on subtomograms containing proteasomes (see Fig. 12). In both processed proteasome sub-tomograms, the contrast has been enhanced and the FSC and cFSC curves provide proof of an improved signal.

**Fig. 13:**
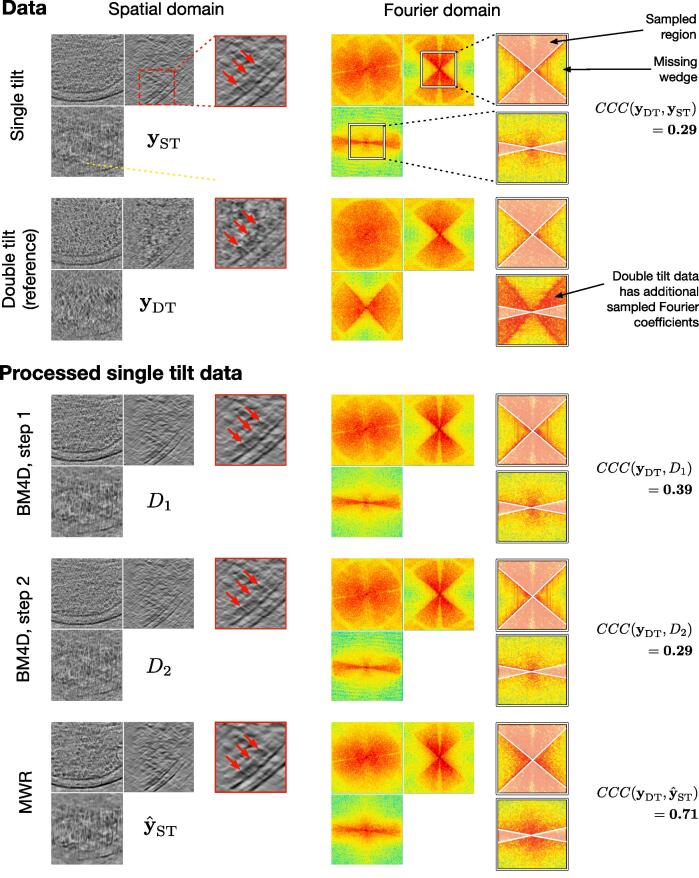
Dataset D: Experimental double-axis sub-tomogram (128×128×128 voxels) containing multiple macromolecules (see red arrows). We process the single-axis version of the volume ($y_{\mathrm{ST}}$) and compare to the double-axis volume ($y_{\mathrm{DT}}$), which acts as a ground truth. We compare the results obtained with BM4D (Step #1 and Step #2) and MWR. All volumes are displayed in spatial domain (left column) and Fourier domain (right column). The thumbnails provide zoomed-in views of the data. For the thumbnails in Fourier domain (right column), the sampled Fourier region has been bleached, in order to focus the attention on the missing wedge. The processed volumes $D_{1},D_{2}$ and y^ST are evaluated by computing the CCC score. $y_{\mathrm{DT}}$ has additional Fourier coefficients yielding signal compared to $y_{\mathrm{ST}}$ (i.e. $y_{\mathrm{DT}}$ has less missing data than $y_{\mathrm{ST}}$). Therefore the CCC score is computed over the support of additional Fourier coefficients. (For interpretation of the references to colour in this figure legend, the reader is referred to the web version of this article.)

Dataset D has a broader field of view than precedent datasets. As can be observed in Fig. 13, the double-axis volume has a better contrast than the single-axis volume, the reason being that it has more sampled Fourier coefficients, and therefore contains more signal and less noise. For this dataset, we processed the single-axis volume and investigated how close to the double-axis volume the restoration results are. We compare the results obtained with the MWR algorithm to those obtained with BM4D (Step #1 and Step #2). From Fig. 13, MWR tends to enhance contrast, which facilitates the identification of membrane and macromolecules. Also, the global contrast is higher in the images restored with MWR than those produced by BM4D. Moreover, we can notice that the output of Step #1 ($D_{1}$ in Fig. 13) has a better constrast than the output of Step #2 ($D_{2}$ in Fig. 13), which is surprising at first glance because Step #2 is generally used to ”boost” the result of Step #1. This suggests that the assumptions made in Step #2 (i.e. additive noise and known stationary signal and noise spectra), do not hold after Step #1 in our specific case in cryo-ET. When examining the data in Fourier domain, it is clear that several Fourier coefficients have been partially recovered, and correlate better with the double-axis volume as confirmed by the CCC values (0.29 before and 0.71 after applying MWR). The volumes obtained with BM4D show little (or no) improvement in terms of CCC values (0.39 for $D_{1}$ and 0.29 for $D_{2}$). All these results confirm that our MWR algorithm improves the restoration of MW when compared to conventional denoising algorithms.

## 6 Conclusion

In this paper, we addressed the problem of restoring an image corrupted by missing Fourier coefficients in a well-localized spectral region (missing wedge). We proposed an original Monte-Carlo method to denoise 3D cryo-ET images and compensate MW artifacts. Our algorithm cannot recover unobserved data, but it merely makes the best statistical guess of what the missing data could be, based on what has been observed. Any non-local or patch-based denoiser can be used in our Bayesian framework, and the procedure converges faster than (Maggioni et al., 2013). Our experiments on both synthetic and experimental cryo-ET data demonstrate that even for high amounts of noise, the method is able to enhance the signal. The method performs better if the contrast of the object of interest is high, which is not always the case in cryo-ET.

In terms of complexity (linear with respect to the number of pixels), the timings of MWR are actually 5.5 min (with a non-optimized version of the algorithm (Matlab) with no parallel implementation) if the number of iterations is set to 500. However, we usually get very similar results after 100 iterations (see Fig. 4), that is 1 min for processing a 64 × 64 × 64 voxels image. To improve computation, several investigations can be performed. We suggest to handle in parallel and fuse several shorter Markov chains (see Louchet et al., 2008, Louchet and Moisan, 2013). Hence, we can exploit multithreading since the Markov chains are independent. At the end, we can expect a gain of factor 10 if we consider only 100 iterations and several Markov chains. For future work, a GPU implementation of the algorithm can be also investigated to process larger volumes.

  **Software** In terms of computational performance, MWR takes 5 min and 30 s (0.66 s/iteration, T=500 samples) on a standard volume of 64×64×64 voxels on a Macbook Pro equipped with 2.9 Ghz Intel Core i7, 16 Gb of RAM and the Mac OS X v.10.12.3 operating system. The computing time increases linearly with the number of voxels. The MRW software (Matlab code) can be downloaded from the Git-Hub website:https://gitlab.inria.fr/serpico/mwr.

  **Data** Datasets B and C have been obtained from tomograms of Chlamydomonas Reinhardtii cells (see (Pfeffer et al., 2017, Albert et al., 2017)] for details about data acquisition). Dataset D has been obtained from a tomogram of E. coli cell, obtained via dual-axis tilt Cryo-ET on FIB-milled samples, and acquired by J. Ortiz (Max-Planck Institute, Chemistry Department, Martinsried, Germany).

  **Author contributions** C.K. conceived the project and designed the research. E.M. developed the algorithm, implemented the code, analyzed and interpreted data. C.K. and E.M wrote the paper, agreed to all the contents and agreed the submission.

## Declaration of Competing Interest

The authors declare that they have no known competing financial interests or personal relationships that could have appeared to influence the work reported in this paper.
